# A novel *Borrelia* species, intermediate between Lyme disease and relapsing fever groups, in neotropical passerine-associated ticks

**DOI:** 10.1038/s41598-020-66828-7

**Published:** 2020-06-30

**Authors:** Florian Binetruy, Stéphane Garnier, Nathalie Boulanger, Émilie Talagrand-Reboul, Etienne Loire, Bruno Faivre, Valérie Noël, Marie Buysse, Olivier Duron

**Affiliations:** 1 MIVEGEC (Maladies Infectieuses et Vecteurs: Ecologie, Génétique, Evolution et Contrôle), Centre National de la Recherche Scientifique (CNRS) - Institut pour la Recherche et le Développement (IRD) - Université de Montpellier (UM), Montpellier, France; 2 UMR 6282 Biogéosciences, CNRS - Université Bourgogne Franche-Comté, Dijon, France; 3 EA7290, Virulence bactérienne précoce, groupe Borréliose de Lyme, Facultés de Médecine et de Pharmacie, Fédération de Médecine Translationnelle de Strasbourg, Université de Strasbourg, Strasbourg, France; 4 French National Reference Center on Lyme borreliosis, CHRU, Strasbourg, France; 5 Unité ASTRE, Centre de Coopération Internationale en Recherche Agronomique pour le Développement (CIRAD), Institut National de la Recherche Agronomique (INRA), UM, Montferriez-sur-Lez, France

**Keywords:** Bacteria, Pathogens

## Abstract

Lyme disease (LD) and relapsing fevers (RF) are vector-borne diseases caused by bacteria of the *Borrelia* genus. Here, we report on the widespread infection by a non-described *Borrelia* species in passerine-associated ticks in tropical rainforests of French Guiana, South America. This novel *Borrelia* species is common in two tick species, *Amblyomma longirostre* and *A. geayi*, which feed on a broad variety of neotropical mammal and bird species, including migratory species moving to North America. The novel *Borrelia* species is divergent from the LD and RF species, and is more closely related to the reptile- and echidna-associated *Borrelia* group that was recently described. Genome sequencing showed that this novel *Borrelia* sp. has a relatively small genome consisting of a 0.9-Mb-large chromosome and an additional 0.3 Mb dispersed on plasmids. It harbors an RF-like genomic organization but with a unique mixture of LD- and RF-specific genes, including genes used by RF *Borrelia* for the multiphasic antigen-switching system and a number of immune-reactive protein genes used for the diagnosis of LD. Overall, our data indicate that this novel *Borrelia* is an intermediate taxon between the LD and RF species that may impact a large host spectrum, including American mammals. The designation “*Candidatus* Borrelia mahuryensis” is proposed for this species.

## Introduction

Bacteria of the genus *Borrelia* (Spirochaetes) are the causative agents of major vector-borne diseases including Lyme disease (LD), the most important tick-borne disease in the northern hemisphere, and relapsing fevers (RF), which are transmitted by ticks and lice worldwide^1,2^. While RF epidemics occurred repeatedly in past centuries^2^, LD is an expanding infectious disease with more than 300,000 new human cases each year in the United States^3,4^. Most LD and RF *Borrelia* species are maintained in enzootic cycles and their presence in humans and domestic animals is incidental to their usual wildlife reservoir hosts^1,2,5^.

The LD and RF *Borrelia* species form two separate sister groups^6,7^, and are sometimes referred to as two sister genera by various authors^8,9^. LD and RF species were usually thought to be the only two groups within *Borrelia*. However, recent surveys have uncovered new *Borrelia* species and strains that do not fall into the LD or RF groups^10–21^. They form a third group, sometimes referred to as “reptile group” (REP), encompassing only two designated species, *B. turcica* and “*Candidatus* Borrelia tachyglossi” (*B. tachyglossi* hereafter), and a few strains not taxonomically described^10–21^. The only two genomes sequenced to date confirmed that these *Borrelia* are substantially different from the LD and RF *Borrelia* groups^19^. Members of the third *Borrelia* group have been detected worldwide, but not in Western Europe^10–14,17,20,21^. They have been detected in hard ticks (*Amblyomma*, *Hyalomma*, *Bothriocroton*, and *Ixodes* genera), which are associated mostly with reptiles, but also with echidna^10–14,20,21^. Recently, new strains of unknown *Borrelia* species, closely related to the third *Borrelia* group, were identified in ticks feeding on ground-dwelling birds (for immature stages) and mammals (for adult stages) in Brazil^16^, Argentina^17^, and the United States^15,17,18^. However, currently, we know little about these new American *Borrelia* strains: Only a small DNA fragment of the flagellin *flaB* gene has been used as an exclusive marker for their identification^15–18^. Unfortunately, this short gene fragment is inadequate for inferring their phylogenetic proximity with known *Borrelia* species since the inner tree topology remains poorly resolved at many nodes because of insufficient sequence polymorphism^15–18^.

In this study, we describe a novel species, belonging to the third *Borrelia* group, in passerine-associated ticks from tropical rainforests of French Guiana. This remote territory is a vast equatorial land located on the north-east coast of South America, mostly covered by dense rainforests and with a low density of human population. This novel *Borrelia* species is common in two tick species feeding on a variety of mammal and bird species. We further cultivated this bacterium and sequenced its complete genome, which showed that this novel *Borrelia* is intermediate between LD and RF *Borrelia*, sharing common features with both of them.

## Results

### A novel *Borrelia* species in passerine-associated ticks

**Figure 1:**
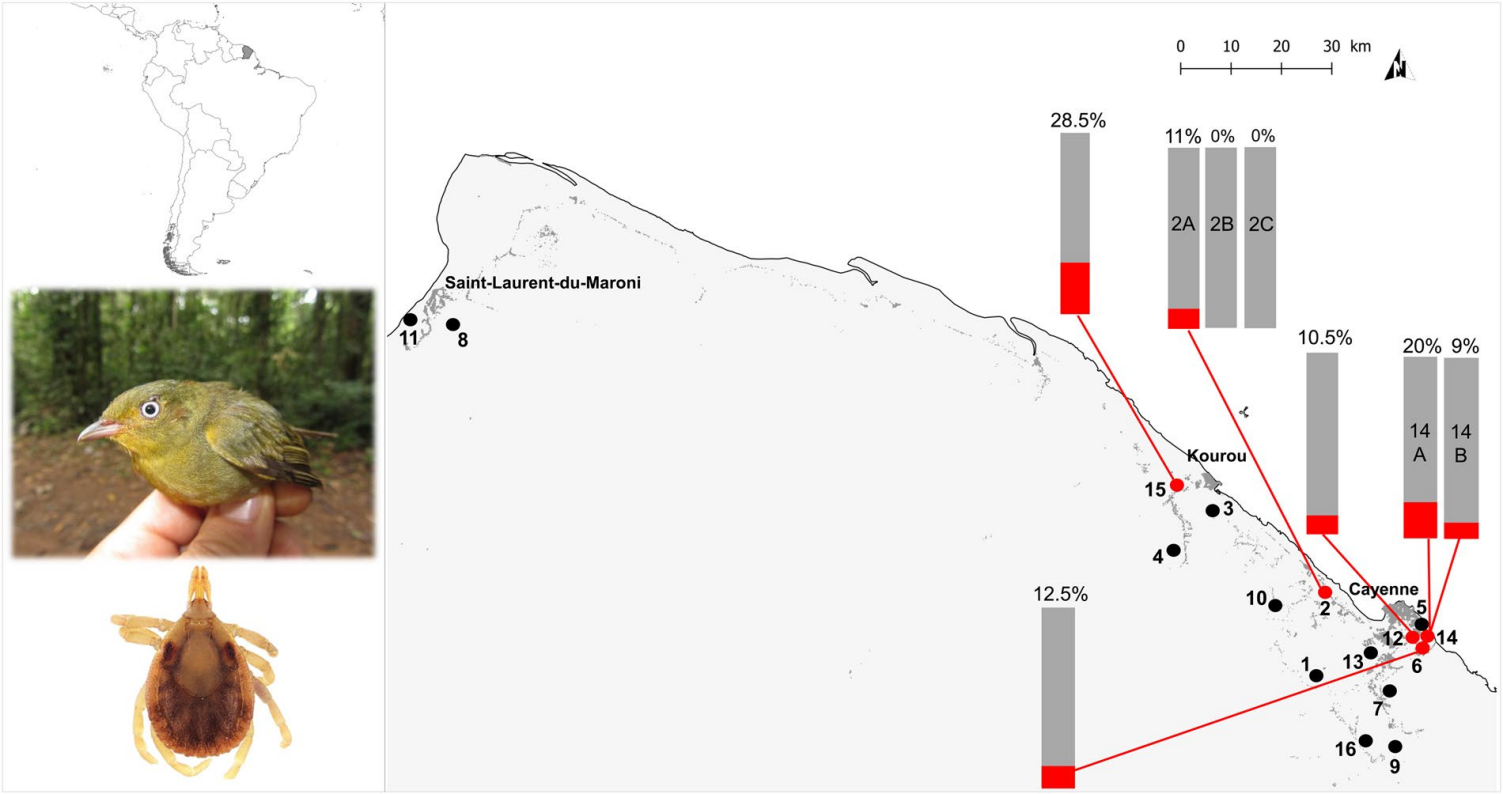
Location of sampling sites in French Guiana. Localities are represented by dots, and numbers correspond to the sampling site number given in Table S1. A letter placed after number indicates a same sampling site sampled over several years as detailed in Table S1. Red and black dots indicate populations infected by *Cand*. Borrelia mahuryensis and populations not infected, respectively. Bar-charts represents the prevalence of *Cand*. Borrelia mahuryensis in the sites where infected ticks were collected. Pictures to the left represents a female Crimson-hooded Manakin (*Pipra aureola*) bearing ticks around its eyes and a nymph of the tick species *Amblyomma longirostre*.

**Figure 2:**
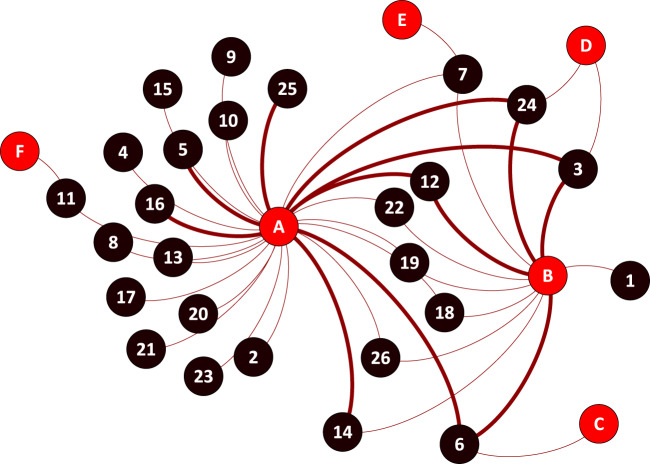
The network of ticks, passerines, and *Cand*. Borrelia mahuryensis. Each red node (with letters) and each black node (with numbers) designate a tick and passerine species, respectively. Solid edges indicate pairs of tick/passerine species for which *Cand*. Borrelia mahuryensis was detected (see Table S1 for details). (**A**) *Amblyomma longirostre*; (**B**) *A. geayi*; (**C**) *A. varium*; (**D**) *A. cajennense*; (**E**) *A. calcaratum*; (**F**) *A*. humerale; 1, *Attila spadiceus*; 2, *Campylopterus largipennis*; 3, *Chiroxiphia pareola*; 4, *Cyanocompsa cyanoides*; 5, *Dendroplex picus*; 6, *Glyphorynchus spirurus*; 7, *Manacus manacus*; 8, *Mionectes macconnelli*; 9, *M. oleaginous*; 10, *Myrmeciza ferruginea*; 11, *Myrmotherula axillaris*; 12, *Percnostola rufifrons*; 13, *Picumnus exilis*; 14, *Pipra aureola*; 15, *P. erythrocephala*; 16, *Ramphocelus carbo*; 17, *Saltator maximus*; 18, *Thamnomanes caesius*; 19, *Thamnophilus doliatus*; 20, *T. punctatus*; 21, *Tolmomyias sulphurescens*; 22, *Turdus albicollis*; 23, *T. fumigatus*; 24, *T. leucomelas*; 25, *Xenops minutus*; 26, *Xiphorhynchus pardalotus*.

A total of 290 tick specimens were collected from 16 sites in French Guiana (from 2012 to 2018), mostly located along the coastline (Table S1 and Fig. 1). Most of the specimens were larvae (*n* = 274), with only few nymphs (*n* = 16), and no adults (Table S1). The 290 ticks were collected from 26 bird species, mainly the blue-backed manakin, *Chiroxiphia pareola* (*n* = 98 ticks; 34%), the wedge-billed woodcreeper, *Glyphorynchus spirurus* (*n* = 75; 26%), and the crimson-hooded manakin, *Pipra aureola* (*n* = 33; 11%). Morphological and genetic examination of tick specimens led to the identification of six tick species, all belonging to the *Amblyomma* genus: *A. longirostre* (*n* = 230), *A. geayi* (*n* = 52), *A. varium* (*n* = 4), *A. cajennense* (*n* = 2), *A. calcaratum* (*n* = 1), and *A. humerale* (*n* = 1). *A. longirostre* was found in 25 bird species, *A. geayi* in 11, and the four other *Amblyomma* species in one or two bird species each (Table S1 and Fig. 2).

Examination of the 290 *Amblyomma* spp. specimens through high-throughput 16 S rDNA sequencing showed that *Borrelia* was present in 20 specimens (6.9%), corresponding to 20 larvae. Infection was detected in 12 of the 230 *A. longirostre* specimens (5.2%) and eight of the 52 *A. geayi* (15.4%) specimens, but not in *A. varium*, *A. cajennense*, *A. calcaratum*, and *A. humerale* (Table S1, Figs. 1 and 2). *Borrelia* was detected in five sampling sites of the 16 examined, and in ticks collected from eight bird species: the woodcreeper *G. spirurus* (three sites), the crimson-hooded manakin *P. aureola* (two sites), the blue-backed manakin *C. pareola* (one site), the pale-breasted thrush *Turdus leucomelas* (one site), the silver-beaked tanager *Ramphocelus carbo* (one site), the straight-billed woodcreeper *Dendroplex picus* (one site), the black-headed antbird *Percnostola rufifrons* (one site), and the white-throated *Xenops minutus* (one site) (Table S1, Figs. 1 and 2). The prevalence of *Borrelia* in *Amblyomma* spp. varied from 0 to 28.5% depending on the sites and sampling dates (Table S1 and Fig. 1), but these variations were not significant (Fisher’s exact tests, all *p* > 0.10).

**Figure 3:**
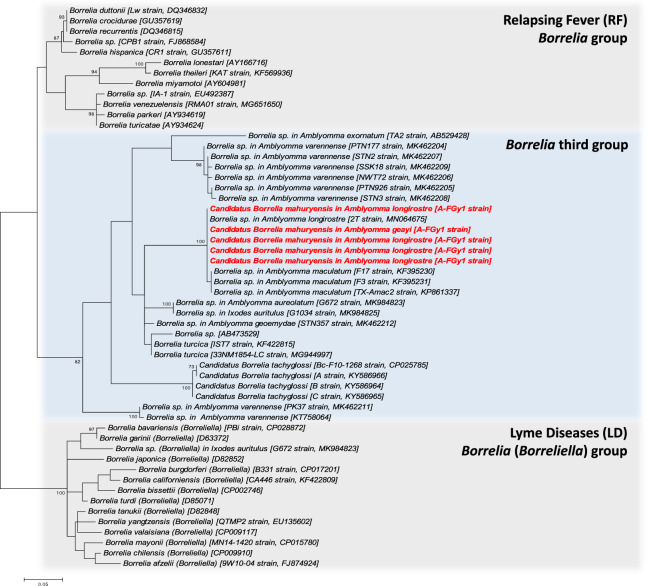
Phylogenetic relationship of *Borrelia* species and strains estimated using maximum likelihood analysis of *flaB* gene sequences (323 unambiguously aligned bp). In red, sequences of *Cand*. Borrelia mahuryensis obtained in this study. Branch numbers indicate percentage bootstrap support (1,000 replicates).

Specific polymerase chain reaction (PCR) surveys of the *Borrelia* 16 S rDNA (710 bp), *flaB* (540 bp), *gyrB* (1172 bp), *groEL* (600 bp), and *glpQ* (784 bp) genes further confirmed the presence of *Borrelia* in the 20 *Amblyomma* spp. specimens earlier found positive by high-throughput 16 S rDNA sequencing. On the basis of these five gene sequences, no nucleotide variation was observed in the *Borrelia* from the *A. longirostre* and *A. geayi* positive specimens, showing that only one *Borrelia* species was present. None of the 16 S rDNA, *gyrB*, *groEL*, and *glpQ* gene sequences observed in this study is 100% identical to other *Borrelia* sequences available on GenBank. However, based on partial *flaB* gene sequences (323 bp), this *Borrelia* strain is 100% identical to the *Borrelia* sp. clone 2 T (GenBank accession number: MN064675) recently found in *A. longirostre* from Brazil^16^. A maximum likelihood (ML) analysis based on these *flaB* gene sequences revealed a robust clustering of the new *Borrelia* of French Guiana with the *Borrelia* sp. clone 2 T of Brazil, along with several other *Borrelia* sp. found in *A. maculatum* of Texas^18^, suggesting that all these *Borrelia* belong to the same species (Fig. 3). ML phylogenetic analysis places this *Borrelia* cluster among members of the third *Borrelia* group, but the inner topology of the *flaB* tree remains too poorly resolved in most cases (as shown by the low support values of the inner branches) to infer the exact relatedness of this *Borrelia* cluster with other *Borrelia* species and groups (Fig. 3).

### Cultivation of *Borrelia*

We obtained a viable *Borrelia* isolate (A-FGy1 hereafter) after 6 weeks of cultivation from a freshly molted tick specimen, obtained from an engorged *A. longirostre* larva collected from a wild passerine. The *Borrelia* A-FGy1 isolate has a typical Spirochaetes helical-shaped structure of ca. 0.2–0.3 μm diameter and 10–25 μm length (Movie S1). The growth rate was slow and the yield of culture was low. Genetic typing revealed no sequence variation in the *flaB*, *gyrB*, *groEL*, and *glpQ* genes between the *Borrelia* A-FGy1 isolated in cultivation and the *Borrelia* typed from tick DNA described above, showing that they belong to the same species. However, there was one single nucleotide polymorphism (SNP) in the 16 S rDNA sequence, suggesting that two closely related *Borrelia* strains are present in the study area. This SNP is upstream the 16 S rDNA V4 hypervariable region we sequenced through high-throughput sequencing, and was thus not detected in our primary bacterial bardocing investigation. However, Sanger sequencing of 16 S rDNA PCR product and Illumina complete genome sequencing consistently show that this SNP is well present in the *Borrelia* A-FGy1 isolate.

### Genomic features of the *Borrelia* A-FGy1 isolate

The sequencing of the *Borrelia* A-FGy1 isolate produced 230,143,188 paired-end reads of 150 bases, which were assembled into 42 contigs with a mean coverage depth of 1,849×(318–7,854×). The final *Borrelia* A-FGy1 genome is 1,236,294 bp in size and consists of a 918,483-bp linear chromosome and 41 contigs of putative plasmids (987–42,459 bp) (Table S2). Overall, the *Borrelia* A-FGy1 genome contains 1,123 genes (812 on linear chromosome and 311 on putative plasmids) including 1,055 predicted protein-coding genes, 37 RNA genes, and 31 pseudogenes. The linear chromosome of the *Borrelia* A-FGy1 isolate had greatest average nucleotide identity (ANI) with members of the third Borrelia group, *B. turcica* (90.2%) and *B. tachyglossi* (82.3%).

**Figure 4:**
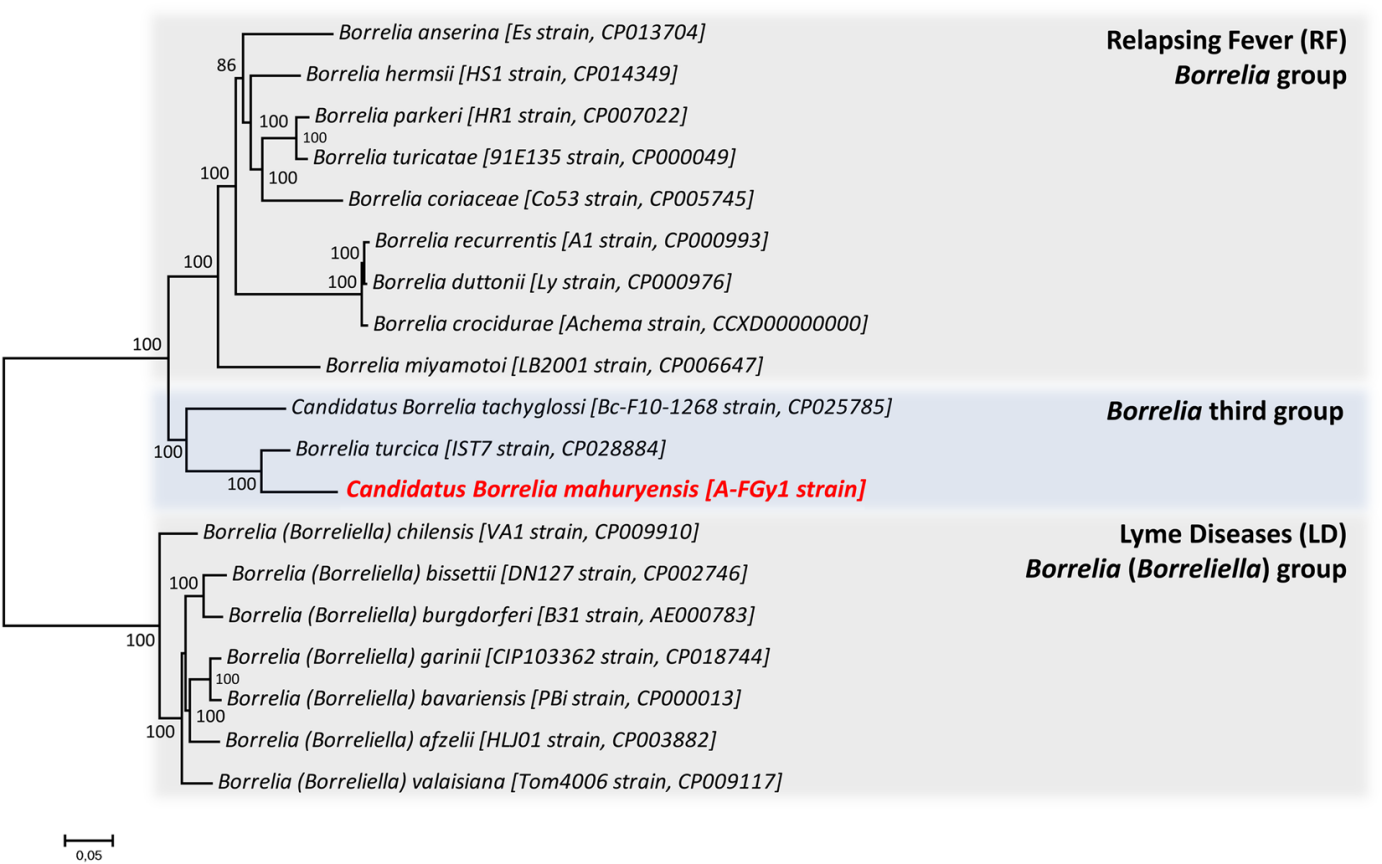
Phylogenetic relationship of 19 *Borrelia* genomes, including the *Cand*. Borrelia mahuryensis A-FGy1 genome (in red). The phylogenetic tree was inferred using maximum likelihood analysis of a concatenated alignment of 590 single-copy orthologous genes (197,675 AA). The numbers on each node represent the support of 1,000 bootstrap replicates.

Phylogenetic analysis based on 590 single-copy orthologous genes (197,675 AA) present in the linear chromosome of 18 other *Borrelia* species showed that the *Borrelia* A-FGy1 isolate clusters within a robust clade with the two known species of the third *Borrelia* group, *B. turcica* and *B. tachyglossi* (Fig. 4). The closest relative of this novel *Borrelia* species is *B. turcica*, which was primarily reported from reptiles and reptile-associated ticks. The third *Borrelia* group, including the *Borrelia* A-FGy1 isolate, clearly forms an independent *Borrelia* lineage. The third *Borrelia* group, however, is more closely related to the RF group than to the LD group (Fig. 4).

The *Borrelia* A-FGy1 genome is roughly similar to the genomes of the two other sequenced members of the third *Borrelia* group, *B. turcica* and *B. tachyglossi*. The *Borrelia* A-FGy1 linear chromosome shows an extensive synteny with chromosomes of other *Borrelia* species and, compared with LD, RF, and the third group of *Borrelia* chromosomes, it exhibits a comparable size and organization (Fig. S1). The single exception concerns a specific 8.5-kb inversion at the 5′ end of the *B. turcica* chromosome but that is absent in the *Borrelia* A-FGy1 chromosome as in other *Borrelia* chromosomes (Fig. S1). Likewise, some *Borrelia* A-FGy1 plasmid contigs were similar in organization to other plasmids common to all *Borrelia* groups: Indeed, the *Borrelia* p6A-FGy1 plasmid contig was largely collinear with linear plasmids of *B. tachyglossi* (lp25), *B. turcica* (lp35), RF species (e.g., *B. miyamotoi* [lpB] and *B. hermsii* [lp53]), and with the circular LD plasmid cp26 (Fig. S2A). Only one *Borrelia* A-FGy1 plasmid contig (p7A-FGy1) was partly collinear with the large linear plasmids (megaplasmids) known exclusively from *Borrelia* species of the third and RF groups. A few plasmid contigs (e.g., p11A-FGy1), however, were highly syntenic with LD, but not RF, *Borrelia* plasmids (Fig. S2B). Another *Borrelia* A-FGy1 plasmid contig, p9A-FGy1, was very similar to the circular plasmid cp33 of *Borrelia turcica* that is absent in all other *Borrelia* species. Several large *Borrelia* A-FGy1 plasmid contigs (e.g., p1A-FGy1, p2A-FGy1, p3A-FGy1, p4A-FGy1 and p5A-FGy1) did not share any common genetic architecture with any previously known *Borrelia* plasmids. However, genes located on these plasmids were homologous to either LD or RF *Borrelia* genes.

**Figure 5:**
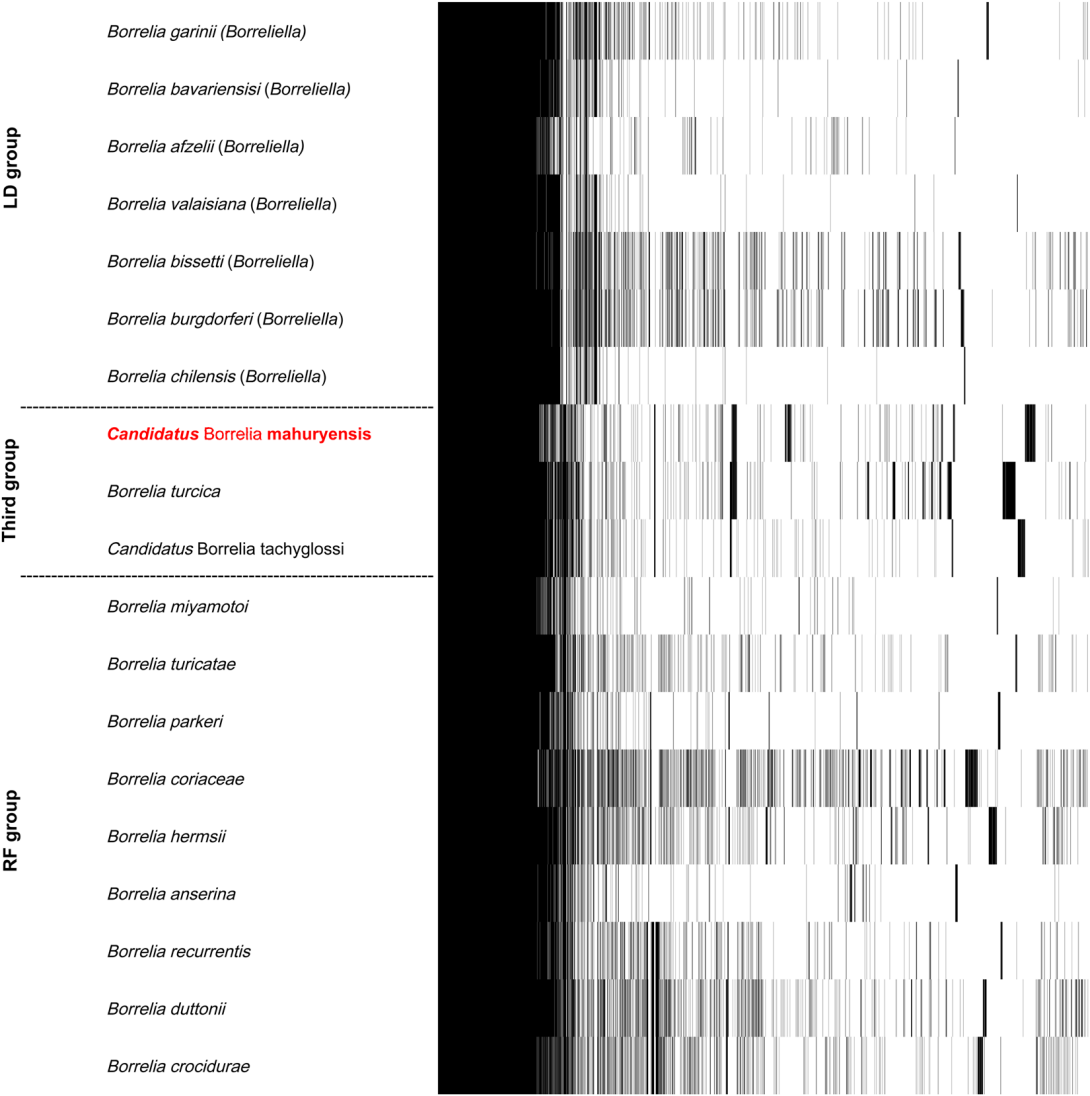
Whole-genome comparison of the pan-genome of the 19 *Borrelia* species and strains used in Fig. 4. Presence of a gene in a genome is indicated in black. The core genome of 638 genes and the genes unique to LD, RF, and third groups are clustered together in the heat map.

Based on gene orthologs, the pan-genome (including the linear chromosome and all plasmids) of third *Borrelia* group members (*B. turcica* and *B. tachyglossi* along with *Borrelia* A-FGy1) shared 806 genes while the core genome of the genus *Borrelia* contained 638 genes, including 590 on the chromosome and 148 on plasmids (Fig. 5). Overall, the third *Borrelia* group genomes shared 8 genes (*kduD*, involved in D-galacturonate catabolic process, *cof*, involved in thiamine biosynthetic process, and 6 hypothetical protein genes) that were not present in the LD and RF *Borrelia* genomes (Table S3). *Borrelia* A-FGy1 however contained 168 unique genes (including a variable large protein *vlp* gene, two iron-sulfur cluster carrier protein genes, one IS200/IS605 family transposase and many hypothetical protein genes), mostly located on plasmids and not present in other *Borrelia* genomes (Table S3). Some genes only present in members of the third *Borrelia* group were absent in *Borrelia* A-FGy1: this includes a pair of genes involved in maltose metabolism (*glvA* and *glvC*) which are inserted within the rRNA operon of *B. turcica* and *B. tachyglossi* but that are absent in *Borrelia* A-FGy1. The *Borrelia* A-FGy1 chromosome also harbors a conserved RF-like gene architecture of the rRNA operon: there is only one copy each of the 23 S rRNA and 5 S rRNA genes (duplicated in LD *Borrelia*), and a horizontally acquired set of three purine salvaging pathway genes (*purA*, *purB*, and *htp*) that are inserted between the 23 S rRNA and 16 S rRNA genes (present in RF but absent in LD *Borrelia* species). The *Borrelia* A-FGy1 genome also contains 19 unique orthologs with *B. tachyglossi*, *B. turcica* and all RF *Borrelia* that are absent in all LD species, including genes involved in important cellular functions such as glycerophospholipid metabolism (*glpT*, *glpQ*) and DNA repair (*RecF*, *RecR*) (Table S3). Finally, the *Borrelia* A-FGy1 genome also contains genes commonly found in LD *Borrelia* genomes but that are absent from all RF *Borrelia* genomes: these genes include the ATP-dependent DNA helicase *PcrA* gene, a tRNA^Met(CAT)^ gene and several hypothetical protein genes (Table S3).

The *Borrelia* A-FGy1 genome harbors a number of previously described genes encoding immune-reactive *Borrelia* proteins, including the Flagellin (*flaB*) and *Borrelia* membrane protein A (*BmpA*) genes located on the linear chromosome and an outer membrane protein OspC homolog gene located on plasmid. Three *Borrelia* plasmid contigs contain variable large protein (vlp) and variable small protein (vsp) genes homologous to those used in the multiphasic antigen variation system of RF *Borrelia* to evade detection by the host immune system. Three *Borrelia* A-FGy1 plasmid contigs contained *vlp* genes: *Borrelia* p6A-FGy1, p33A-FGy1, and p35A-FGy1, which contained one *vlp* gene each. One *vsp* gene was identified on the *Borrelia* p6A-FGy1 plasmid contig and shared high homology to *B. turcica vsp35*, *B. hermsii vsp24*, *B. turicatae vspB*, and *OspC* from LD *Borrelia* species.

### Proposal of candidate name

On account of these distinct and coherent microbiological, phylogenetic, and evolutionary traits described above, we propose the designation “*Candidatus* Borrelia mahuryensis” for this novel bacterium associated with the passerine-associated ticks *A. longirostre* and *A. geayi*. The specific name refers to Mount Mahury, French Guiana, which was the first sampling site where we detected the presence of this bacterium.

## Discussion

We found that a novel *Borrelia* species, *Cand*. Borrelia mahuryensis, divergent from the LD and RF *Borrelia* species, is common in passerine-associated ticks in tropical rainforests of French Guiana. We obtained a pure culture isolate of *Cand*. Borrelia mahuryensis (A-FGy1 isolate) that is morphologically similar to those of other *Borrelia* species. This novel *Borrelia* species is more closely related, although distinct, to the two known species of the third *Borrelia* group, *B. turcica* and *B. tachyglossi*. At the five loci extensively examined, only one SNP was observed between all our *Cand*. Borrelia mahuryensis DNA samples, showing that at least two very closely related strains are circulating in French Guiana. The recent detection of *Borrelia* isolates in Brazil^17^ sharing the same *flaB* sequence with *Cand*. Borrelia mahuryensis, along with the presence of closely related strains in Texas^18^, suggests that *Cand*. Borrelia mahuryensis may have a broad geographic distribution across American countries, with a substantial intraspecific variation. Finally, examination of the *Cand*. Borrelia mahuryensis A-FGy1 genome confirmed its difference from other *Borrelia* species, but revealed the presence of shared features with either LD or RF or both *Borrelia* species.

The repeated detection of *Cand*. Borrelia mahuryensis from 2012 to 2018 confirms that infection persists durably in French Guiana through its circulation in at least two tick species, *A. longirostre* and *A. geayi*. Infected tick specimens were collected from eight passerine species, suggesting that these vertebrates can be natural hosts for *Cand*. Borrelia mahuryensis. Interestingly, the related *Borrelia* strains detected in Brazil were also detected in *A. longirostre*^16^ collected from ground-dwelling birds, and those detected in the United States were detected in *A. maculatum*^15,18^, a species that also parasitizes birds among other hosts^22,23^. All of this evidence supports the hypothesis of birds as natural hosts for *Cand*. Borrelia mahuryensis. However, *A. longirostre*, *A. geayi*, and *A. maculatum* are also known to be associated with a variety of vertebrate species, with the immature stages usually feeding on passerine birds and the adults feeding instead on arboreal mammals such as new world porcupines and sloths (for *A. longirostre* and *A. geayi*)^24,25^ or large mammals such as cattle (for *A. maculatum*)^22,23^. Natural hosts of *Cand*. Borrelia mahuryensis can thus be either passerine birds, or mammals, or both. The observation that tick larvae are commonly infected suggests the existence of two non-exclusive scenarios: (i) passerine birds are the hosts, with tick larvae acquiring *Borrelia* through feeding on infected passerine birds; (ii) mammals are the hosts, with tick females acquiring *Borrelia* through feeding on infected mammals and further transmitting the infection transovarially to their larvae. Transovarial transmission, however, is not a common feature in the *Borrelia* genus since it has been demonstrated often for RF, but rarely for LD, species^26–29^. Interestingly, the isolation of *Cand*. Borrelia mahuryensis in culture from a freshly molted tick demonstrates that infection is maintained through molting to subsequent developmental stages in the tick host. Thus, through this transstadial transmission, infected ticks have the potential to infect animals on which they subsequently feed, including humans on whom *A. longirostre* and *A. maculatum* can occasionally feed^22,23,30,31^. Furthermore, while both *A. longirostre* and *A. geayi* are native to South and Central America^24,32–34^, migratory birds also regularly introduce *A. longirostre* to North America^32,35–39^. Bird migration (for *A. longirostre*, *A. geayi*, and *A. maculatum*) and cattle transportation (for *A. maculatum*) may be two important factors affecting the distribution of *Cand*. Borrelia mahuryensis over long distances and across geographical barriers, thereby explaining its wide geographic distribution.

The genome of *Cand*. Borrelia mahuryensis is more similar to those of the *Borrelia* third group, *B. tachyglossi* and *B. turcica*, which were also recently sequenced^19^. Members of this group shared similar gene content, including specific genes not present in LD or RF *Borrelia*. When compared with LD or RF *Borrelia* species, the genome of *Cand*. Borrelia mahuryensis shows more similarities with RF *Borrelia* species and harbors genes not found in LD *Borrelia*. However, it also harbors LD-specific genes and plasmids, showing that *Cand*. Borrelia mahuryensis exhibits intermediate features between the RF and LD groups. The *Cand*. Borrelia mahuryensis genome encodes several immunogenic vlp and vlp proteins (used in the multiphasic antigen variation system of RF *Borrelia* to evade detection by the host immune system^40^) as well as a number of immune-reactive proteins, including flab and BmpA, which are used for the diagnosis of LD^41^. Altogether, these genomic features suggest that *Cand*. Borrelia mahuryensis and other members of the third *Borrelia* group form a continuum of *Borrelia* species between the LD and RF groups, affecting our ability to clearly distinguish between these two groups.

To conclude, the description of *Cand*. Borrelia mahuryensis shows that the third *Borrelia* group is both widespread and biologically diverse. Most of the known members of this group were found in association with reptile hosts^10,13,14,20,21^, with the exception of one echidna host^11,12^. However, the description of *Cand*. Borrelia mahuryensis now suggests that members of the third *Borrelia* group have a large host spectrum that may also include a variety of birds and mammals as natural hosts. It is perhaps not unexpected to find other members of the third *Borrelia* group infecting a wide diversity of vertebrates. They have RF-like genomes with several housekeeping and macronutrient metabolism genes only present in the RF *Borrelia* species. Paralogous vlp and vsp proteins playing key roles in the RF multiphasic antigenic variation system and pathogenicity are also conserved, but *Cand*. Borrelia mahuryensis also have LD-specific orthologs. Future studies should give pivotal clues about the biology of *Cand*. Borrelia mahuryensis, as recently done through *in vitro* experiments with other members of the third *Borrelia* group: Indeed, variable levels of *B. turcica* resistance to vertebrate serum suggest that tortoises are reservoir host species while birds or humans are not^42^. Additional studies of *Cand*. Borrelia mahuryensis are needed to determine its transmission cycle and to establish whether these bacteria are pathogenic for birds and mammals, including humans.

## Material and methods

### Study area and tick sampling

Birds were captured using mist nets during the dry season (2012–2018) at 16 sites within forest patches (Table S1 and Fig. 1). Before releasing the birds, ticks were collected with fine forceps and immediately stored in 75% ethanol until examination and molecular screening (*n* = 290). An additional batch of ticks (*n* = 19) were collected from the Rémire-Montjoly site (site 14B in Table S1) and were taken alive to the laboratory for *Borrelia* cultivation (see below). All ticks were morphologically identified to species level using morphological and genetic diagnostic criteria^25^. The global connectivity between tick and bird species was visualized using the network analysis software package Gephi^43^.

### Molecular screening and typing

Tick whole-body DNA was extracted using an extraction kit according to the manufacturer’s instructions (Qiagen). The presence of *Borrelia* was further examined through DNA barcoding involving the production of PCR amplicons from a 251-bp portion of the V4 variable region of the bacterial 16 S rDNA using a Multiplex PCR Kit (Qiagen) and universal primers (16 SV4F: 5′-GTGCCAGCMGCCGCGGTAA-3′ and 16SV4R: 5′-GGACTACHVGGGTWTCTAATCC-3′)^44^ as previously described^45^. Amplified bacterial 16S rDNA products were purified and sequenced using an Illumina MiSeq platform (GenSeq, Montpellier University) and 250-bp end sequence reads were obtained. All bioinformatic analyses were conducted using the pipeline Frogs^46^ as previously described^45^. Sequences with 97% similarity were clustered together and identified as an operational taxonomic unit (OTU). Each representative OTU sequence was aligned and taxonomically assigned using the Silva database (https://www.arb-silva.de/).

Independent PCR assays for *Borrelia* species identification were performed through the amplification of *flaB*, *gyrB*, *groEL*, *glpQ*, and 16 S rRNA gene fragments using specific primers (Table S4). All PCR products were visualized via electrophoresis in a 1.5% agarose gel. Positive PCR products were purified and sequenced in both directions using Sanger method (Eurofins). Sequence chromatograms were manually cleaned with CHROMAS LITE (http://www.technelysium.com.au/chromas_lite.html), and alignments were performed using CLUSTALW^47^, implemented in the MEGA V7 software^48^.

### *Borrelia* cultivation

Engorged tick specimens (*n* = 19) were taken alive to the laboratory for blood meal digestion. Ticks were kept in humidified chamber (80–90% relative humidity) until molting (only three remained after molting: one female and two nymphs). Freshly molted ticks (*n* = 3) were further surface-sterilized with a bleach solution, rinsed with PBS before being individually cut into two parts. The two parts of the ticks were transferred together into a 6 ml-tube to isolate borreliae that was further cultured at 34 °C in BSK-II modified medium (6% rabbit serum, 6% gelatin, 30 µg rifampicin) in anaerobic conditions^49^ for several weeks and regularly examined under dark-field microscopy. Presence of *Borrelia* in remnants of the ticks was investigated through a specific PCR assay targeting the *flab* gene (using primers listed in Table S4).

### Genome sequencing and analyses

One *Borrelia*-positive culture was used to prepare DNA-seq libraries using the Illumina Nextera DNA Flex sample preparation kit. Library validity was assessed by quantification using a Fragment Analyzer and a Qubit (Invitrogen). DNA-seq experiments were performed on an Illumina MiniSeq via a platform (MGX, Montpellier) using Illumina MiniSeq Mid Output Reagent Cartridge with a paired-end read length of 2×150 bp. A total of 6 GB of data were obtained. The quality of Illumina reads was analyzed with FastQC (http://www.bioinformatics.babraham.ac.uk/projects/fastqc/) and reads were further cleaned and trimmed using Trimmomatic^50^. The remaining reads were assembled into contigs and then into scaffolds with the SPAdes v3.8 assembler^51^. The annotation was performed via the NCBI prokaryotic genome annotation pipeline^52^. Dot plot analyses were done using YASS^53^. Whole-genome alignments were performed using Mauve^54^.

Pangenomic analysis (presence/absence of genes in the 19 complete genomes) was conducted with prokka^55^ and roary^56^. Roary was run with default parameters that are well tailored to infer of the most accurate number of genes^56^. Paralogs were then split according to the authors methodology. Briefly, if multiple genes from the same sample were found in an orthologs cluster, the neighborhood −5 genes up and downstream - of putative paralogs are used to infer information about synteny and split the cluster accordingly. This can be problematic for mis-assembled contigs or for multigenic family repeated in tandem, but allow genes with similar sequence to be placed in different cluster if they lie in different genomic position.

### Molecular phylogenetic analyses

For analyses of single gene sequences, the GBLOCKS program^57^ with default parameters was used to remove poorly aligned positions and to obtain unambiguous sequence alignments. Closely related organisms obtained from GenBank were also included in the analyses. The evolutionary models that best fit the sequence data were determined using the Akaike information criterion with the program MEGA v7^48^. Tree-based phylogenetic analyses were performed using maximum likelihood (ML) analyses. ML heuristic searches using a starting tree obtained by neighbor joining were conducted in MEGA v7^48^. Clade robustness was assessed by bootstrap analysis using 1,000 replicates.

A phylogenomic approach was followed using ITEP^58^. All other complete *Borrelia* genomes were obtained from GenBank. Multiple orthologs were aligned with MAFFT^59^ v7. The concatenated multiple alignment was cleaned with trimal. The phylogenetic tree was computed by RaxML v8.2.4^60^ using an ML approach with a GAMMA-LG model and 1,000 bootstrap replicates.

### Ethics approval

All animals were handled in strict accordance with good animal practice as defined by the French code of practice for the care and use of animals for scientific purposes, established by articles R214-87 to R214-137 of the French rural code. All captures were performed by competent people without causing avoidable pain, suffering, distress, or lasting harm to the birds. Bird sampling was done under permission granted by several organizations: the Direction de l’Environnement, de l’Aménagement et du Logement (DEAL) from Guyane, the Direction Régionale de l’Office National des Forêts (ONF) de Guyane, the Conservatoire du Littoral (CEL) de Guyane, the Collectivité Territoriale de Guyane (CTG), the Centre National d’Etudes Spatiales (CNES), the Centre Spatial Guyannais (CSG). The use of the genetic resources was declared to the French Ministry of the Environment (reference TREL1902817S/156), in compliance with the Access and Benefit Sharing procedure implemented by the Loi pour la Reconquête de la Biodiversité.

## Supplementary information

Supplementary information.

Movie S1. Microscopy movie of the culture of Cand. Borrelia mahuryensis A-FGy1. Three motile bacteria are apparent in this low-density culture medium.

Table S1.

Table S3.

Table S4.

Table S5.

## Data Availability

The genome of *Cand*. Borrelia mahuryensis A-FGy1 has been deposited at GenBank under the accession number #SAMN12690807.
