# Supplementary material for: A novel Borrelia species, intermediate between Lyme disease and relapsing fever groups, in neotropical passerine-associated ticks
Source: Sci Rep. 2020 Jun 30;10:10596. doi: 10.1038/s41598-020-66828-7 (PMC7327063; doi:10.1038/s41598-020-66828-7)
Supplement: Supplementary file 1 — Supplementary information. [file 41598_2020_66828_MOESM1_ESM.docx]

**A novel *Borrelia* species, intermediate between Lyme disease and relapsing fever groups, in neotropical passerine-associated ticks**

Florian Binetruy^1^, Stéphane Garnier^2^, Nathalie Boulanger^3-4^, Émilie Talagrand-Reboul^3-4^, Etienne Loire^5^, Bruno Faivre^2^, Valérie Noël^1^, Marie Buysse^1^, Olivier Duron^1^*

^1^ MIVEGEC (Maladies Infectieuses et Vecteurs : Ecologie, Génétique, Evolution et Contrôle), Centre National de la Recherche Scientifique (CNRS) - Institut pour la Recherche et le Développement (IRD) - Université de Montpellier (UM), Montpellier, France

^2^ UMR 6282 Biogéosciences, CNRS - Université Bourgogne Franche-Comté, Dijon, France

^3^ EA7290, Virulence bactérienne précoce, groupe Borréliose de Lyme, Facultés de Médecine et de Pharmacie, Fédération de Médecine Translationnelle de Strasbourg, Université de Strasbourg, Strasbourg, France.

^4^ French National Reference Center on Lyme borreliosis, CHRU, Strasbourg, France

^5^ Unité ASTRE, Centre de Coopération Internationale en Recherche Agronomique pour le Développement (CIRAD), Institut National de la Recherche Agronomique (INRA), UM, Montferriez-sur-Lez, France

* Correspondence: [olivier.duron@ird.fr](mailto:olivier.duron@ird.fr)

**SUPPLEMENTARY MATERIALS**

**Table S1.** List of tick species and populations included in the analysis, with details of their origin, the population sample size (*n*), and the prevalence of *Cand.* Borrelia mahuryensis.

**Table S2.** Summary of *Candidatus* Borrelia mahuryensis A-FGy1 chromosome and plasmid assemblies.

| Sequence | Size (bp) | Coverage | GenBank |
| --- | --- | --- | --- |
| *Candidatus* Borrelia mahuryensis A-FGy1 |  |  |  |
| Chromosome | 918,483 | 515,41 | CP043682 |
| Plasmid p1A-FGy1 | 42,459 | 393,21 | CP043683 |
| Plasmid p2A-FGy1 | 27,186 | 735,92 | CP043684 |
| Plasmid p3A-FGy1 | 25,747 | 3940,09 | CP043685 |
| Plasmid p4A-FGy1 | 21,951 | 1642,93 | CP043686 |
| Plasmid p5A-FGy1 | 21,815 | 661,97 | CP043687 |
| Plasmid p6A-FGy1 | 21,789 | 1843,09 | CP043688 |
| Plasmid p7A-FGy1 | 18,775 | 830,19 | CP043689 |
| Plasmid p8A-FGy1 | 17,174 | 317,95 | CP043690 |
| Plasmid p9A-FGy1 | 11,219 | 706,94 | CP043691 |
| Plasmid p10A-FGy1 | 10,999 | 1335,40 | CP043692 |
| Plasmid p11A-FGy1 | 9,905 | 1393,59 | CP043693 |
| Plasmid p12A-FGy1 | 8,769 | 1117,30 | CP043694 |
| Plasmid p13A-FGy1 | 8,255 | 1397,39 | CP043695 |
| Plasmid p14A-FGy1 | 7,784 | 876,51 | CP043696 |
| Plasmid p15A-FGy1 | 6,149 | 847,17 | CP043697 |
| Plasmid p16A-FGy1 | 6,148 | 1743,44 | CP043698 |
| Plasmid p17A-FGy1 | 4,157 | 2787,78 | CP043699 |
| Plasmid p18A-FGy1 | 4,087 | 1639,13 | CP043700 |
| Plasmid p19A-FGy1 | 3,34 | 667,99 | CP043701 |
| Plasmid p20A-FGy1 | 3,144 | 7854,36 | CP043702 |
| Plasmid p21A-FGy1 | 3,118 | 1947,38 | CP043703 |
| Plasmid p22A-FGy1 | 2,79 | 4688,99 | CP043704 |
| Plasmid p23A-FGy1 | 2,517 | 2052,96 | CP043705 |
| Plasmid p24A-FGy1 | 2,387 | 2215,40 | CP043706 |
| Plasmid p25A-FGy1 | 2,344 | 2108,29 | CP043707 |
| Plasmid p26A-FGy1 | 2,162 | 2841,64 | CP043708 |
| Plasmid p27A-FGy1 | 2,099 | 1379,75 | CP043709 |
| Plasmid p28A-FGy1 | 1,975 | 1781,40 | CP043710 |
| Plasmid p29A-FGy1 | 1,875 | 1332,41 | CP043711 |
| Plasmid p30A-FGy1 | 1,827 | 1626,07 | CP043712 |
| Plasmid p31A-FGy1 | 1,611 | 600,25 | CP043713 |
| Plasmid p32A-FGy1 | 1,502 | 1738,87 | CP043714 |
| Plasmid p33A-FGy1 | 1,423 | 1760,29 | CP043715 |
| Plasmid p34A-FGy1 | 1,395 | 6466,83 | CP043716 |
| Plasmid p35A-FGy1 | 1,38 | 644,93 | CP043717 |
| Plasmid p36A-FGy1 | 1,205 | 1499,74 | CP043718 |
| Plasmid p37A-FGy1 | 1,161 | 699,90 | CP043719 |
| Plasmid p38A-FGy1 | 1,138 | 4539,66 | CP043720 |
| Plasmid p39A-FGy1 | 1,042 | 1162,78 | CP043721 |
| Plasmid p40A-FGy1 | 1,021 | 622,14 | CP043722 |
| Plasmid p41A-FGy1 | 987 | 2706,91 | CP043723 |

**Table S3.** List of gene presence and absence in the pan-genome of 19 *Borrelia* species, including *Candidatus* Borrelia mahuryensis A-FGy1. Presence of a gene in a genome is indicated in black in the table, with the corresponding source Gene ID.

**Table S4.** Genes and primers used for *Borrelia* screening and multilocus typing.

| **Genes** | **Hypothetical product** | **Gene position on the Borrelia A-FGy1 chromosome** | **Primers (5'-3')** |  | **Tm** | **Fragment size** | **References** |
| --- | --- | --- | --- | --- | --- | --- | --- |
| *16S rRNA* | Small ribosomal subunit | 446626-448167 | Borr16SF1- | CGAAGAGTTTGATCCTGGCTTAG | 54°C | Semi-nested PCR assay: | This study |
|  |  |  | Borr16SR1- | CGTGAYTCAGCGTCAGTCTTGACC |  | 1st round PCR: Borr16SF1/Borr16SR2: 1506bp |  |
|  |  |  | Borr16SR2- | CAGTACGGCTACCTTGTTACGAC |  | 2nd round PCR: Borr16SF1/Borr16SR1: 757bp |  |
| *gyrB* | DNA gyrase subunit B | 456171-458075 | BorrgyrBF1- | ATGARTTATGTTGCTAGTAA | 48°C | Semi-nested PCR assay: | This study |
|  |  |  | BorrgyrBR1- | CTCTCHGAYTCTCTTGCTT |  | 1st round PCR: BorrgyrBF1/BorrgyrBR2: 1208bp |  |
|  |  |  | BorrgyrBR2- | CCAGCAGAATCWCCTTCTACAAT |  | 2nd round PCR: BorrgyrBF1/BorrgyrBR1: 1172bp |  |
| *glpQ* | Glycerophosphodiester phosphodiesterase | 249407-250411 | BorrglpQF1- | ATAGCTCAYAGAGGTGCHAG | 48°C | Semi-nested PCR assay: | This study |
|  |  |  | BorrglpQR1- | TTACATAWGAAGGYAATGC |  | 1st round PCR: BorrglpQF1/BorrglpQR1: 808bp |  |
|  |  |  | BorrglpQF2- | TATTTACCAGAACATACHTTAG |  | 2nd round PCR: BorrglpQF2/BorrglpQR1:: 784bp |  |
| *groEL* | Chaperone protein GROEL | 694274-695914 | BorrgroELF1- | GATGCTCTTGCTGCDCTTGTT | 50°C | Semi-nested PCR assay: | This study |
|  |  |  | BorrgroELR2- | ACATCATTCCCATTCCWGGAT |  | 1st round PCR: RatpAF1/RatpAR2: 624bp |  |
|  |  |  | BorrgroELR1- | TCCCATTCCWGGATCCATAGG |  | 2nd round PCR: RatpAF2/RatpAR2: 600bp |  |
| *flaB* | Flagellin B | 145704-146714 | BorrflaBF1- | CTGATGATGCTGCTGGNATGG | 54°C | Nested PCR assay: | This study |
|  |  |  | BorrflaBR2- | TGTGCACCATCTTGCTGCTGAG |  | 1st round PCR: BorrflaBF1/BorrflaBR2: 545bp |  |
|  |  |  | BorrflaBR1- | CGCTTGAGCTGGTGATTCTGCTA |  | 2nd round PCR: BorrflaBF1/BorrflaBR1: 518bp |  |

**Figure S1.** Dot plot view of the aligned chromosomes of *Cand.* Borrelia mahuryensis A-FGy1 with *B. turcica* (GenBank accession number: CP028884), *B. tachyglossi* (CP025785), *B.* *miyamotoi* (CP006647), and *B. burgdorferi* (AE000783). The chromosomes show high conservation of global gene order with only one genomic rearrangement at the end of the *B. turcica* chromosome (red asterisk).

**(A)**

**(B)**

**Figure S2.** Physical map of (**A**) the *Borrelia* p6A-FGy1 plasmid and plasmids from *B. tachyglossi*, *B. turcica,* and various LD and RF *Borrelia* species, (**B**) the *Borrelia* p9A-FGy1 plasmid and plasmids from various LD *Borrelia* plasmids. Each contiguously colored locally collinear block (LCB) represents a region without rearrangement of the homologous backbone sequence estimated with the Mauve aligner. Lines between genomes indicate orthologous LCBs. LCBs below the center in *Borrelia* genomes represent blocks in the reverse orientation.

**Movie S1.** Microscopy movie of the culture of *Cand.* Borrelia mahuryensis A-FGy1. Three motile bacteria are apparent in this low-density culture medium.
